# From Laser Scanning to Finite Element Analysis of Complex Buildings by Using a Semi-Automatic Procedure

**DOI:** 10.3390/s150818360

**Published:** 2015-07-28

**Authors:** Giovanni Castellazzi, Antonio Maria D’Altri, Gabriele Bitelli, Ilenia Selvaggi, Alessandro Lambertini

**Affiliations:** Department of Civil, Chemical, Environmental, and Materials Engineering (DICAM), University of Bologna, V.le Risorgimento 2, Bologna 40136, Italy; E-Mails: antoniomaria.daltri@studio.unibo.it (A.M.D.); gabriele.bitelli@unibo.it (G.B.); ilenia.selvaggi2@unibo.it (I.S.); alessandro.lambertini@unibo.it (A.L.)

**Keywords:** terrestrial laser scanning, historical buildings, geometric modeling, finite element analysis, structural analysis, cultural heritage

## Abstract

In this paper, a new semi-automatic procedure to transform three-dimensional point clouds of complex objects to three-dimensional finite element models is presented and validated. The procedure conceives of the point cloud as a stacking of point sections. The complexity of the clouds is arbitrary, since the procedure is designed for terrestrial laser scanner surveys applied to buildings with irregular geometry, such as historical buildings. The procedure aims at solving the problems connected to the generation of finite element models of these complex structures by constructing a fine discretized geometry with a reduced amount of time and ready to be used with structural analysis. If the starting clouds represent the inner and outer surfaces of the structure, the resulting finite element model will accurately capture the whole three-dimensional structure, producing a complex solid made by voxel elements. A comparison analysis with a CAD-based model is carried out on a historical building damaged by a seismic event. The results indicate that the proposed procedure is effective and obtains comparable models in a shorter time, with an increased level of automation.

## 1. Introduction

A terrestrial laser scanner (TLS) is an optical instrument [1,2] that allows the generation in near real-time fine geometric representation of objects by means of accurate dense clouds of 3D points. This technology, following a systematic pattern, captures in a three-dimensional space the external surface of a physical object. The instrument may deliver additional information, such as reflectivity values or even color. Despite its high cost, TLS is used today to perform numerous tasks: the sensor allows acquiring, in a short time, the current condition of the surveyed object and obtaining a snapshot of its geometry.

This concept has been applied very often within the field of cultural heritage to support multidisciplinary studies: from simple documentation, to monitoring the condition of historical buildings and also in order to support restoration works or structural analysis checks. The 3D model allows storing metric and qualitative information for further use. The possibility of having a complete coverage of a building changes the traditional concept of a survey, where only the data of some defined sections are available [3,4].

A further benefit arises from the possibility to integrate the geometric and radiometric information, in order to produce textured 3D models and also an orthophoto derived from digital pictures and the 3D survey. They can support, for instance, the analysis of some degenerations of the building condition, such as the one produced by the presence of moisture content.

Without being limited to the external façades, TLS can be also used to survey internal parts of the building. This can be applied, for instance, for damage assessment in post-earthquake surveys, where the damage of buildings is widespread. Thereby, the present study aims at introducing a new semi-automatic procedure to generate a finite element (FE) model from a laser scanner survey of a whole building.

The problem of the automatic transformation of a large point cloud dataset to a simplified geometrical object is a well-known and studied topic. Several contributions are available in the literature, and some of them propose semi-automatic procedures specialized for a specific use. In the context of an airborne laser scanner (ALS), the automatization aims at reconstructing simple shapes of buildings to use with textures from terrestrial and airborne images. Consider, for instance, [5], where an approach for the automated generation of building models from ALS, comprising the entire sequence from extraction to reconstruction and regularization, is presented, or [6], where the 3D simplified modeling of buildings is finalized to a study about solar radiation potential. Recently, in the field of civil engineering, laser scanner surveys are gaining particular interest to generate structural models, since the increasing computational capabilities allow the manipulation of large datasets. The automation aims at reconstructing precisely the building’s complexity by its main geometric features. Although there are several studies available in previous literature, in some cases, the cloud is simple or significantly simplified. In [7], a pipeline to reconstruct the complete geometry of architectural buildings from point clouds obtained by sparse range laser scanning is presented for buildings that are made of planar faces. The proposed technique faithfully constructs a polyhedron of low complexity based on the incomplete scans, but does not resolve fine geometry details. In [8], a three-dimensional cloud of points is used to generate a cross-section model that is applicable to structural analysis, while in [9], a finite element method (FEM) analysis of a whole building is carried out using laser scanning data. In [10], a methodology to estimate the deformation of arches or vaults based on the symmetry of sections obtained along the vault guideline is presented. In this case, the accurate geometry of the masonry arches is obtained by means of a three-dimensional laser scanner survey, reduced to the inner arches’ surface representation. Massive structures, such as masonry bridges, can also be investigated by summing the laser scanner survey information with those obtained by ground penetrating radar, obtaining a fine picture of the external and internal feature [11]. Here, the cloud simplification lies in the sampling of some points useful for reconstructing the geometry by means of regular geometry.

A highly relevant contribution to this field is proposed in [12,13], where an attempt to precisely capture the building geometry by automatic reconstruction of its boundary is performed. Moreover, in the contribution given in [14], a point-based voxelization method to automatically transform point cloud data into solid models for computational modeling is presented. The method constructs a triangular irregular network (TIN) mesh by means of a voxel grid bounding the cloud region. The resulting model captures the three-dimensionality of the survey, but does not capture the whole structure, since it is designed for façades [12,13].

Regarding the reconstruction of buildings from point clouds, it is possible to update existing 3D models with accurate information acquired from TLS [15]. In [16], a laser scanner survey is combined with radar interferometric modeling, in a multidisciplinary context, in order to produce an FE model of an ancient masonry tower to study the dynamic behavior.

An interesting approach is presented in [17,18], where the authors describe the use of building information modeling (BIM) derived from point clouds for the structural simulation based on FEM. They observe that although BIM interoperability has reached a significant level of maturity, the density of laser point clouds provides very detailed BIM models that cannot directly be used in FE software. In fact, in order to achieve the expected FE model, several manual corrections are needed to guarantee the mesh compatibility, to avoid mesh local distortions or small elements and to model complex architectural objects.

In this paper, we present and validate CLOUD2FEM, a new semi-automatic procedure to transform three-dimensional point clouds of complex objects into a three-dimensional finite element model. The procedure conceives of the point cloud as a stacking of point sections. The point cloud used in our case study for the procedure validation was collected by performing a laser scanning survey.

Our procedure requires starting with a previously-acquired dataset: consider, for instance, within the cultural heritage scope, a survey that aims at documenting a historical building, supporting restoration works or allowing simple direct structural evaluation.

The complexity of the clouds is arbitrary, since the procedure is designed for terrestrial laser scanner surveys of buildings with irregular geometry, such as historical buildings. It should be stressed, however, that this procedure does not require a particular methodology of acquisition of the cloud, which is therefore not limited to TLS, but can be obtained using digital photogrammetry or any other suitable technique to define the geometry of the detected object [19].

As previously said, the TLS data output can reach a high level of detail and has to be synthesized to produce data input for FE modeling. This operation is not trivial, because the simplified model must retain all of the information regarding the structural elements. The proposed method always guarantees the generation of a filled model ready to use for structural analysis purpose.

The paper is organized as follows: Section 2 presents the proposed procedure and illustrates its potential by means of a simple application; Section 3 presents the validation of the procedure by its application to a complex historical monumental building: a structural analysis of the building is presented and discussed by comparing the finite element model obtained with the proposed procedure with a CAD-based finite element model. Some concluding remarks end the paper.

## 2. Proposed Method

Given an accurate description of a complex geometry, our procedure allows the reconstruction of the original three-dimensional geometry by means of a particular discretization.

In order to apply the procedure, some preliminary (common) operations may be required in order to improve the reconstruction quality and to reduce the error propagation due to the registration of large and complex point clouds. The point clouds can rarely be directly transformed into a filled and complete 3D model without user intervention: the complex shape of the geometry, the irregularly distributed spatial points and the missing faces are critical aspects that usually prevent the automation of the process.

### 2.1. CLOUD2FEM Conceptual Workflow

**Figure 1:**
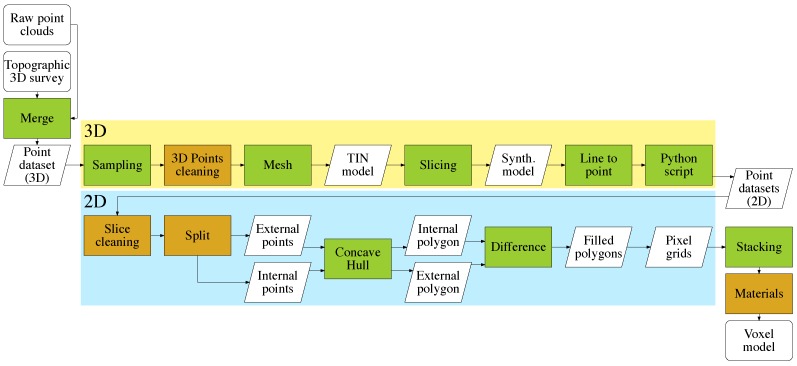
Flowchart for the proposed method: completely automated procedures (green) and semi-automated or manual procedures (orange).

The procedure followed in our case study is synthesized in the flowchart of Figure 1. The input data consists of generic point clouds, merged into a single point data file for the whole building surveyed. The workflow begins with a 3D analysis divided into sequential steps. Most of the operations here described are completely automated (highlighted in green in the flowchart) using different algorithms. At the end of this first part, the building is described with a dataset of slices, each containing bi-dimensional points. These are subsequently analyzed in a 2D environment individually, and this phase includes some semi-automatic or manual analysis (highlighted in orange in the flowchart). All data are georeferenced using a unique local or global reference system; therefore, final datasets are stackable. Each pixel grid, obtained from the corresponding slice, contributes to the creation of the voxel model.

### 2.2. Slice Generation from the Point Cloud Survey

The laser scanner is one of the three-dimensional active measurement systems. In particular, ranging scanners are used for architectural surveys, thanks to their direct measurement of the distance up to hundreds of meters. They are divided into time of flight (TOF) and phase comparison measurement.

In the first method, a laser pulse is sent to the object, and the distance between the transmitter and reflecting surface is computed from the travel time derived from signal transmission and reception. Scanners use rotating devices, such as a mirror or a prism, for the angular deflection of the laser beam. With the phase comparison method, the beam is modulated by a harmonic wave. The distance in this case is calculated by comparing the phase difference between the emitted and the received wave. Due to the more complex signal analysis, the results may be more accurate, but with a reduced range [3].

A ranging scanner is very efficient: it can record hundreds of thousands of points per second and can achieve precision in the order of a few millimeters on the coordinates of the single points [20].

A typical TLS survey is composed of several stages. The first is the planning, which depends on the specific object to survey. It determines the various positions of the instrument (scan positions), based on the good overlap between scans in order to avoid undetected areas and to obtain a proper density according to different parameters. Two laser scanner specifications are critical to the detection of object features in the point cloud: the smallest possible change in angle between two successive points and the size of the laser spot on the object [21]. From a user’s point of view, the resolution has to be selected in order to yield the smallest detail to be detected, to avoid the phenomena of oversampling that may introduce noise into the model.

The following phase is to properly position the targets, used as tie points in common between adjacent scan positions, in order to merge all of the point clouds acquired in a unique three-dimensional model. It is possible to use the well-known ICP (iterative closest point) algorithm, which can connect point clouds without using individual tie points, starting from approximately-positioned and -oriented point clouds [19]. In a survey of a large complex building, with an accurate three-dimensional topographic survey georeferencing each target, it is possible to reduce the error propagation derived from registering together a large number of point clouds.

After having fulfilled all of the previous requirements, it is possible to acquire the data with the TLS instrument, in the various scan positions previously determined. At the same time, it is useful to obtain optical images as an integration of the point clouds. Software is then used to process the data surveyed and to align and merge all of the point clouds, applying a filter to eliminate any noise in the final point cloud. The robustness of the alignment can be enhanced using known coordinates from the acquired targets, producing a unique, optimized point cloud.

**Figure 2:**
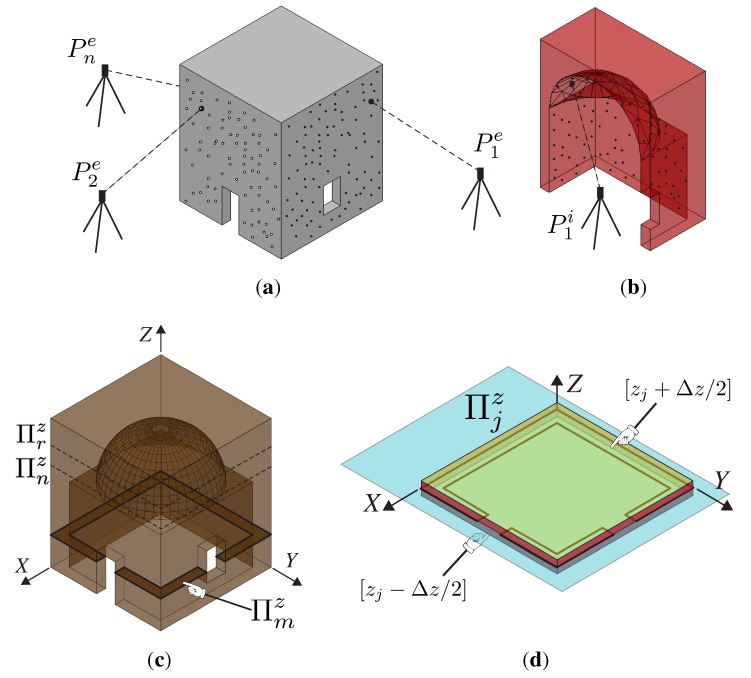
Visualization of the stacking layer sequence concept. (**a**) Point cloud survey of external façades; (**b**) point cloud survey of internal surfaces; (**c**) illustration of the *m*-th slice; (**d**) ${\text{Π}}_{j}^{z}$ layer.

Point cloud slicing is a common procedure to extract sections and details from large point cloud databases. CAD-based procedures are often used to transform sliced points into line-based models using automated procedures based on segmentation or using a manual extraction of profiles; see, forinstance, [22] regarding the processing of building façades. Upon this condition, we conceive of the point cloud as a stacking layer sequence of “planar points”. In Figure 2, a simple geometry is illustrated and referenced to the Cartesian system, where the axis Z is the principal direction of the stacking sequence (Figure 2c). The structure is subdivided by subsequent section planes ${\text{Π}}_{j}^{z}$, each one characterized by an incremental *z*-coordinate of Δz. Then, all of the points within the range $\left[z_{j}-\text{Δ}z/2,z_{j}+\text{Δ}z/2\right]$ are projected to the mid-plane ${\text{Π}}_{j}^{z}$ (see Figure 2d).

Therefore, it is possible to reduce a three-dimensional problem to a two-dimensional problem. By means of a parsing algorithm that walks through the dataset and separates points belonging to each slice, it is possible to generate a number of two-dimensional layers describing the whole structure when they are stacked together. This model is composed from slices containing only points with variable (x,y) coordinates and a constant $z_{j}$ coordinate. The distance between two adjacent slices has to be chosen according to the desired final resolution of the FE model.

It is possible to operate on each slice independently with effective techniques with a linear workflow using software for the management of spatially-referenced data (e.g., a GIS). The pattern of points located on the ${\text{Π}}_{j}^{z}$ plane is generated from the points belonging to each section of Δz thickness. A sequence of points at a constant interval and high density is placed along the lines contained in each slice. A boundary polygon that encloses the points can be computed using a concave or convex hull algorithm [23].

In the case of a building, the slices contain two principal profiles: the first made by connecting the points that belong to the external point cloud (consider, for instance, the survey of the external façade of a building; see Figure 2a) and the second made by connecting the points that belong to the internal point cloud (consider, for instance, the survey of the internal rooms of a building; see Figure 2b).

The first result in a filled geometry we call external, because it envelops the whole building. This might also be composed of several islands that represent the outside face of the building walls.

Similarly, the second produces a boundary polygon called internal that is computed selecting only the points acquired in the internal rooms. It is important to emphasize that also this polygon, in the same way as the external one, is created using a concave hull algorithm [24], enveloping the point selection from the outside. Additionally, this polygon may also be composed of several islands representing the various rooms of the building.

Now, we have two polygons, one external and one internal, and both have a filled geometry. By subtracting the second from the first, we obtain our first result: a filled polygon for each slice of the building that describes the entire structure.

### 2.3. Finite Element Model Generation from Slices

**Figure 3:**
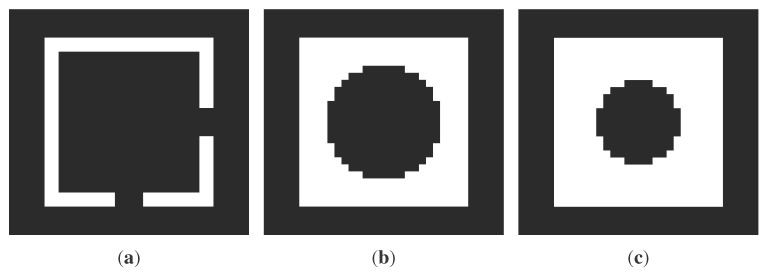
Two dimensional images obtained by slicing the structure illustrated in Figure 2: *m*, *n* and *r* represent three generic slices located at the *z_m_*, *z_n_* and *z_r_* coordinates respectively. (**a**) ${\text{Π}}_{m}^{z}$; (**b**) ${\text{Π}}_{n}^{z}$; (**c**) ${\text{Π}}_{r}^{z}$.

Once the slices have been created, we need to introduce the discretization procedure in order to set up the desired FE model. Thereby, we propose to discretize first the two-dimensional sections and then to use them to build the three-dimensional discretized model. By using the computed tomography (CT) approach, each slice has been idealized as a digital image, with a certain resolution, composed of picture elements (pixels), so the stacking of these slices generates the volume elements (voxels). This procedure allows the reconstruction of the original three-dimensional geometry by stacking all of its slices, so a complete volumetric representation of the object is obtained by acquiring a contiguous set of slices. Consider, for instance, the work in [25], where a dynamic structural analysis has been performed within the biology field using the CT scan dataset directly. The original polygon is then described by a N×M pixel matrix corresponding to a grid of pixels with a particular resolution. With reference to Figure 2c and Figure 3, the pixel value will be, for instance, in an eight-bit grayscale, 255 for the filled area and zero for empty spaces. This transformation is performed automatically for each slice with a fixed region preserving the output resolution. Therefore, all of the grids are aligned, and we build the voxel model stacking them in the original order, following their coordinates. It is worth noting that the voxels are not placed along the internal and external profiles as proposed by other techniques [12,13,14].

**Figure 4:**
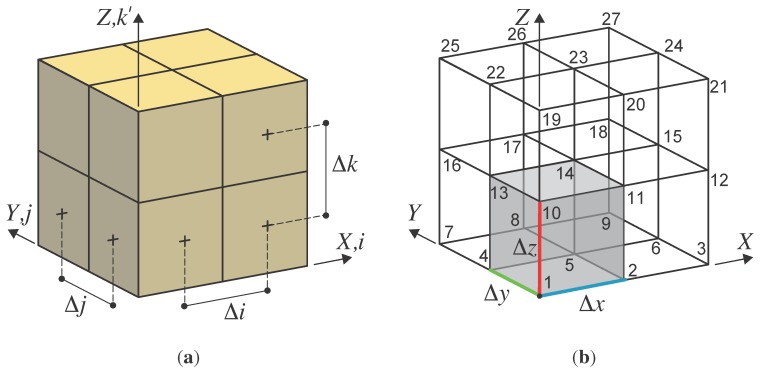
Voxel representation and finite element transformation: {*i*, *j*, *k*} and {*X*, *Y*, *Z*} are the indexes of the voxels’ three-dimensional matrix and the global coordinates of the structure, respectively. The coordinate *k*' means *k*' = *R* − *k*, where Ris the third size (along *Z*) of the voxels’ three-dimensional matrix (*N* × *M* × *R*). (**a**) Voxel indexes;(**b**) hexahedral elements.

Voxels, as represented in Figure 4a, define a particular grid structure that possesses thefollowing features:

- ${\text{Π}}_{j}^{z}$ planes are chosen with the normal along Z that is the building construction direction: features, layers and openings are conceived of by a stacking of elements (*i.e.*, bricks) along the Z direction;
- Δz is chosen according to the building complexity along the Z direction;
- Δx and Δy are chosen according to the in-plane complexity and are totally independentof Δz;
- The stacking procedure is here proposed as a linear stacking of contiguous slices, but can be, in general, considered as interpolated along the Z direction (*i.e.*, considering more slices at a time);
- The resulting discretized volume does not need any particular further adjustment “to fill” the structure.

The resulting dataset is simple and easy to use with the finite element technique: each voxel is automatically transformed into an eight-node hexahedral finite element. By using a common space-partitioning data structure (KD-TREE), the scheme represented in Figure 4a is transformed into a finite element structure (Figure 4b) by simply generating the connectivity structure of each element.

In theory, this operation can be performed for each voxel value (in this case, zero or 255) or only for a certain value of the voxel, *i.e.*, only those with a value equal to 255. Therefore, it is possible to easily describe multiple properties of objects by setting multiple values for the voxel. For instance, if we assume that a particular voxel value corresponds to a particular material, we can describe, in addition to the geometry, also multiple mechanical properties.

With these features, the resulting discretized geometry already contains all of the information to use with the FE model, including the mechanical properties associated with the material features. The proposed method guarantees, with a simple procedure, the construction of a fine discretized geometry and, then, an automatic generation of a reliable FE solid model. Since we are dealing directly with the definition of finite element nodal coordinates and with the connectivity matrix, the proposed method is generally customized to work with any commercial FE software. This rational organization is certainly the key novelty introduced by the method.

**Figure 5:**
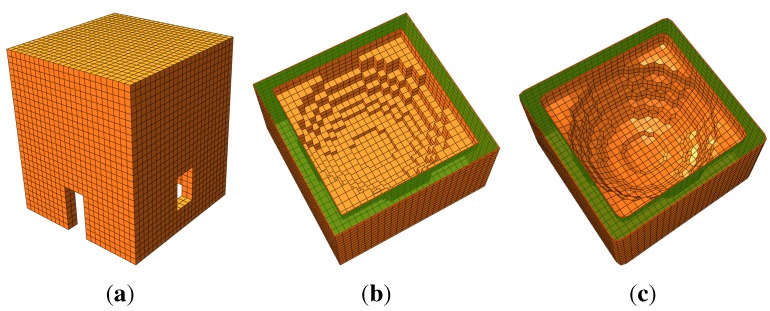
Finite element mesh obtained by applying the procedure to the structure represented in Figure 2. (**a**) External restitution; (**b**) internal restitution; (**c**) smoothed internal restitution.

Figure 5 illustrates the FE mesh obtained by applying the procedure to the structure represented in Figure 2. As can be noticed, as long as the surface is regular and parallel to the axis directions, the resulting mesh precisely matches the original geometry (Figure 5a), but when the surface is irregular (curved) or not planar to an axis direction, the resulting FE mesh is a jagged representation of the original geometry (Figure 5b). Despite this fact, it is always possible to improve the mesh accuracy using a smoothing method to reduce the faceting; see Figure 5c. Concisely, these methods are linear low-pass filters that remove high curvatures variations (jag) and have to be chosen in order to not produce shrinkage; see, for instance, [26]. In what follows, we do not use any of those procedures, since we are interested in testing the raw procedure.

## 3. Procedure Validation: The Case of the San Felice sul Panaro Fortress

In order to validate and show the capabilities of the proposed technique, the case of the San Felice sul Panaro Fortress is presented and discussed.

### 3.1. The San Felice sul Panaro Fortress

The San Felice sul Panaro Fortress is a monumental historical building located near the city of Modena, in San Felice sul Panaro (Italy). In 2012, it was hit by the Emilia earthquake with two magnitude peaks on 20 May ($M_{W}$ = 5.86) and on 29 May ($M_{W}$ = 5.66) [27], and it is the object of several studies that aim to preserve its integrity [28,29]. As the first intervention, the municipality of San Felice did a fine survey of the damaged building by using geomatic techniques, mainly laser scanning and photogrammetry, obtaining different products, like point clouds, orthophotos and immersive visualization.

**Figure 6:**
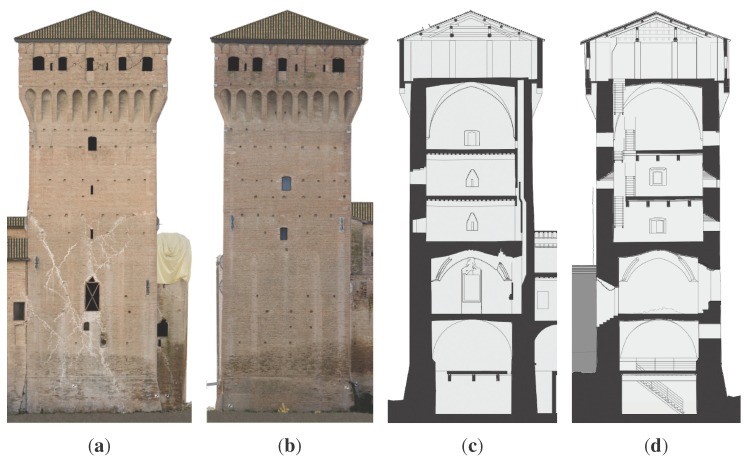
San Felice sul Panaro Fortress principal tower. (**a**) South front; (**b**) east front; (**c**) E-W section; (**d**) S-N section.

This paper focuses attention on the principal tower (Mastio) with the aim to extensively test the capabilities of this new technique. As shown in (Figure 6), the tower is composed of six layers of different kinds: cross-vaults, wood slabs with old and remodeled structures. Each level is then characterized by irregular dimensions and thickness. By inspecting the south front illustrated in Figure 6a, it is shown how the seismic shock hit and damaged the tower by producing a lateral and torsional oscillation and residual displacements on the actual configuration. Openings are placed irregularly on the structure and also have irregular shapes and sections (Figure 6c,d). Summing up, the structure is anything but regular.

### 3.2. Fortress Laser Scanner Survey and Slice Generation

A morphological and structural survey for the San Felice sul Panaro Fortress was planned by request of the Municipality in order to generate a functional representation of the actual state of the building. The survey was performed by ABACUS s.a.s., using a FARO Focus 3D × 330 laser scanner [20] and a total station Trimble S6. The building has a complex structure due to its construction, which took place in several stages over several centuries, forming an irregular geometry. The analysis has become more complex after the earthquake, because of the presence of debris in some interior rooms. Numerous targets were then placed, for precise identification of correlation points between scans, for both the exterior and the interior of the fortress. A closed polygonal topographic network was prepared, to detect the position of each target using the total station. This network has been properly calculated and compensated. Subsequently, 163 point clouds have been acquired by different scanning positions using the laser scanner. These scans are aligned to the topographic network through correlation with the reference targets, resulting in millimetric precision. Using the software Gexcel JRC 3D Reconstructor, following the first decimation, the aforementioned clouds were merged into a unique cloud containing more than 40 million points.

In order to simplify this initial point cloud, a particular algorithm [30] was used to populate a new dataset with a point sampling generated according to a Poisson-disk distribution (sampling procedure in Figure 1), using the open source mesh processing system MeshLab [31]. The result was a new point cloud reduced to 3.2 million points, with a regular spatial sampling of 0.050 m, suitable for further analysis. The next operation was to clean the point cloud (3D point cleaning in Figure 1), mainly removing all neighbor points not belonging to the building of interest. In fact, other surrounding buildings were acquired during the initial scan, in order to align all of the different point clouds. These buildings can be removed from the point cloud, reducing it further down to 1.9 million points.

**Figure 7:**
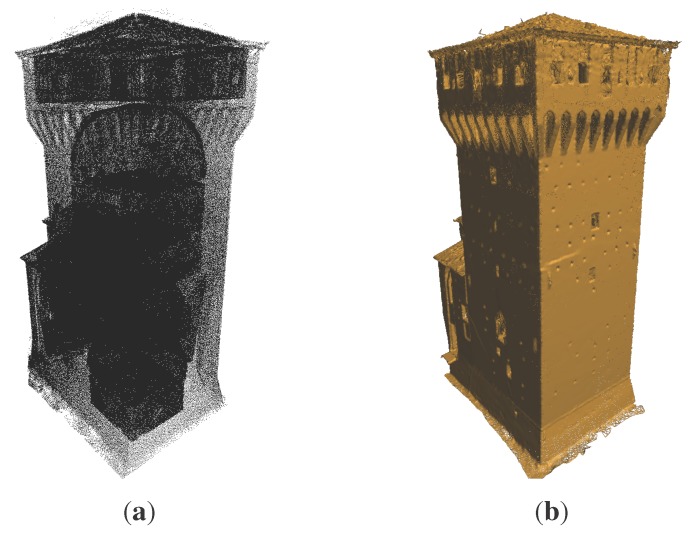
Three-dimensional models for Mastio tower. (**a**) Points; (**b**) triangular irregular network (TIN) mesh.

**Figure 8:**
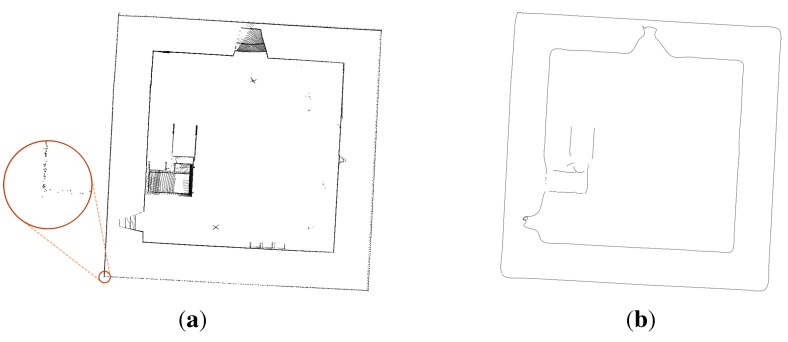
First part of the slicing workflow (3D data): the magnified portion shows the uneven density of the raw data. (**a**) Points within the Δ*z* increment; (**b**) final processed slice of points.

From the point cloud, a subset of 0.8 million points related to the Mastio has been extracted and analyzed; see Figure 7a. As represented in Figure 7b, the survey finely describes every single feature of the structure. The point cloud has a very heterogeneous density, primarily related to the distance between the single scan positions and the object acquired. Figure 8a represents the points that are located within the range $\left[z_{j}-\text{Δ}z/2,z_{j}+\text{Δ}z/2\right]$, at a given $z_{j}$ coordinate. In Figure 8a, the lower left corner is magnified to show the original point cloud density.

The special conditions of the building must be considered. Regarding the exterior part of the model, there were problems with the roofs that were partially collapsed, and these were covered with large plastic tarpaulins in order to avoid water infiltration. Furthermore, in the surroundings and in the internal courtyard of the building, there was piles of rubble and debris. All of these elements hide the actual geometry of the building from the laser scanner point of view. In fact, in the three-dimensional point cloud, these elements are acquired and then intrinsically fused with the proper model of the building, and there is no automatic procedure to perform a full cleaning in advance.

The point cloud is a three-dimensional model. However, it is necessary to build a model that consists of continuous surfaces in order to define the exterior and interior shell of the structure (mesh and polygonal model in Figure 1), through TIN mesh or non-uniform rational B-spline (NURBS) surfaces. After having obtained the shell of the structure, it is possible to define the volume of the structure to be filled with voxels following the other steps, which will be listed later. Directly creating a voxel model from the point cloud, the first result would be only the voxel corresponding to the points detected by the scanner, that is only a surface model and not a volume model. In order to create a volume model, the structure would need to be filled with more voxels, having to solve non-trivial problems for areas in shadow with respect to scan positions. In correspondence to these positions, the data are missing; therefore, holes remain in the model that are not then possible to fill. We avoid this problem by analyzing the model slice by slice. In this study, the polygonal model has been realized using the TIN mesh (Figure 7b). Considering the points (x,y,z) in space, the conjunction between them is realized with lines forming adjacent triangles in order to represent the object with a continuous surface.

The mesh consists of a total of 4.8 million triangles. This model describes all of the surfaces surveyed with the laser scanner, but it cannot be considered a correct closed model from the topological point of view; consider, for instance, the roof surface in Figure 7.

Regarding the interior part of the model, we must account for the fact that, at the moment of the laser scanner acquisition, there was furniture in different rooms, as well as rubble and debris in some areas. Every disturbing element increases the complexity of the building, as illustrated in Figure 8.

By inspecting every single slice in GIS software (in this case, QGIS has been used), it appears very easy to find and properly clean every slice from points that do not belong to the building, but have been inevitably acquired during the scanning. Using our procedure, by creating a concave hull that envelopes the internal points from the outside, the presence of internal debris or any furniture located inside the room is irrelevant, because each new shape is based on the peripheral points. This operation is fundamental to obtain a closed shape for each slice, directly using the geometry provided from the previous step, without any smoothing.

This part of the procedure is semi-automatic. We believe that some manual intervention is essential at this stage for an accurate separation between internal points and external points: this is especially true with data from complex buildings, such as the one analyzed. The proposed workflow aims at minimizing manual intervention in terms of time in order to maximize the efficiency of the procedure itself.

Based on the Mastio geometry properties, we consider that a fine description of the tower can be done by slicing the tower height with a Δz=0.200 m, which corresponds, more or less, to three layers of bricks and two layers of mortar. On the other hand, the resolution of each slice is set to have Δx=Δy=0.115 m, which corresponds to the short dimension of the brick (half-brick).

**Figure 9:**
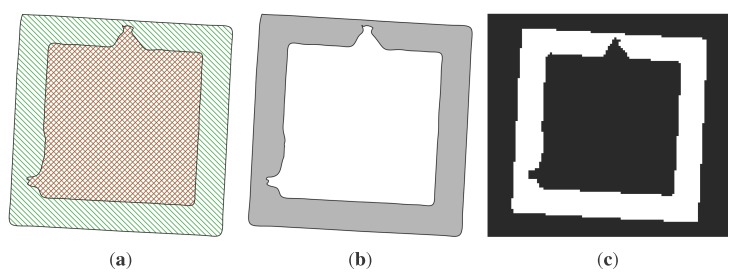
Second part of the slicing workflow (2D data). (**a**) Internal (red), external (green); (**b**) filled slice; (**c**) bitmap: 116 × 107 pixels.

The resulting stacking sequence is composed of 153 horizontal slices, where each one is represented by a N×M grid of 116×107 pixels. Figure 8 and Figure 9 illustrate the *i*-slice representative of a generic section.

### 3.3. Mastio Finite Element Model Generation

**Figure 10:**
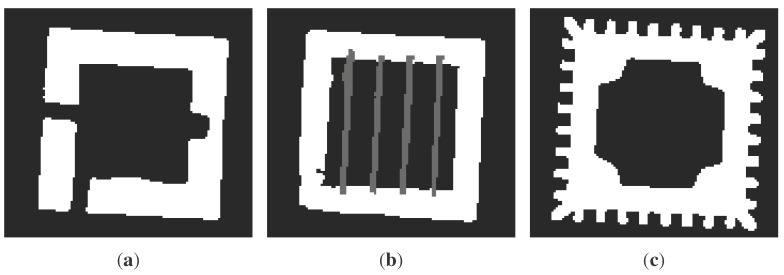
Examples of bitmap slices. (**a**) 42nd slice; (**b**) 84th slice; (**c**) 128th slice.

**Figure 11:**
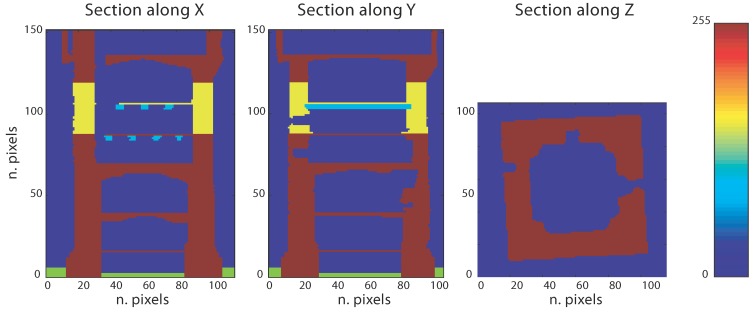
Visualization of the three-dimensional material matrix: voxels possess unitary dimension. Five materials (colors) are used to represent the structure according to the mechanical characterization given in Table 1.

Thanks to the material properties’ survey, the structure has been entirely described by using five different material properties, the mechanical properties of which are set according to [32,33]; see Table 1. Figure 10 describes the generic section representation where the user can visualize and set the material properties based on his knowledge, which might have been acquired from direct inspection or available images. The resulting three-dimensional matrix is visualized by plotting its pattern by means of RGB colors in Figure 11.

**Table 1:** Mechanical characterization of the materials by color.

Material	Color	Elastic Modulus	Poisson’s Coefficient	Density
	(0–255)	(MPa)	(-)	(kg/m${}^{3}$)
**Masonry**	255	1500	0.20	1800
**Reinforced Masonry**	150	1900	0.20	1800
**Terrain**	125	–	–	–
**Timber**	100	8000	0.37	415
**Air**	0	–	–	–

Voxels are plotted by transforming row and column indexes to a unitary coordinate. Then, the generation of the eight-node hexahedral FE model is done according to the instructions given in Figure 4 by associating their coordinates respectively to the Δx, Δy, Δz volume. It is important to notice that the three-dimensional matrix contains volumes for any arbitrary index (i,j,k) combination, *i.e.*, values are also assigned to empty spaces (surrounding air, terrain, *etc*.). The user can choose, according to the FE model purposes, to filter out some of the values. For instance, here, the voxels corresponding to the air and terrain properties are excluded by not being processed during the mesh generation procedure.

**Figure 12:**
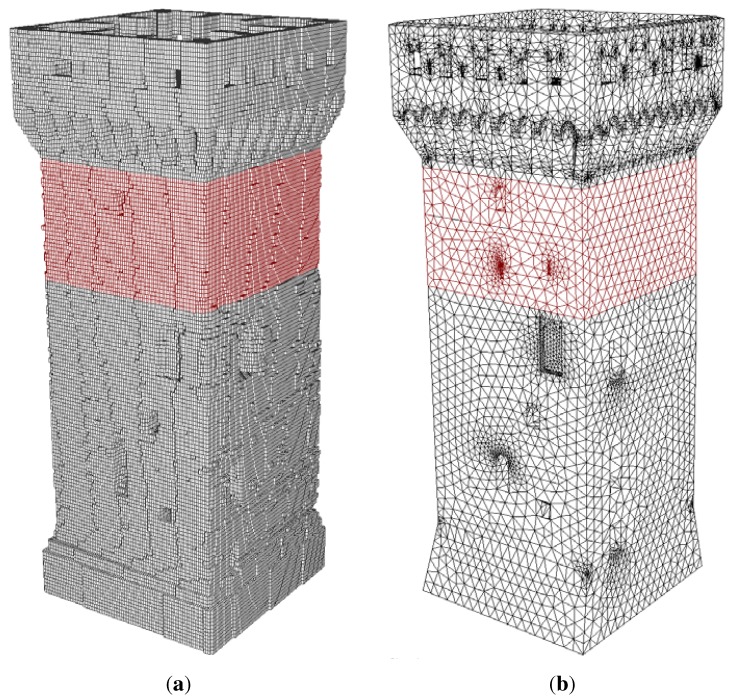
Finite element discretization comparison; colors are set according to the material properties: gray and red colors are used to illustrate the masonry and the reinforced masonry elements, respectively. (**a**) CLOUD2FEM discretization; (**b**) CAD-based discretization.

The final mesh is then characterized by 745,668 nodes and 661,105 elements; see Figure 12a.

### 3.4. Structural Analysis and Comparison with the CAD-Based Model

The finite element model obtained by using the proposed procedure is tested within a structural analysis. A comparison is performed using a very accurate finite element model obtained through a precise CAD procedure based on the same laser scanner dataset; see Figure 12. The referencemodel [29] is obtained by means of tetrahedral four-node elements to model the masonry walls and four-node shell elements to model vaults and layers, counting 54,340 nodes and 215,938 elements.

In order to assess the accuracy of the proposed model, a linear natural frequency analysis (eigenvalue analysis) is performed. Clamped boundary conditions have been considered for nodes located at the ground level (Z=0).

The linear natural frequency analysis is a common tool for the characterization of structures’ dynamic behavior and also used for historical masonry structures [16,34,35,36]. The natural frequencies and the natural mode shapes of vibration, which are the characteristics of the structure, are given by the solution of the following eigenvalue problem:

KΦ=λMΦ

where M is the mass matrix, K is the stiffness matrix, λ is an eigenvalue and Φ is its relative natural mode shape of vibration (eigenvector). The eigenvalue problem does not fix the absolute amplitude of the vector Φ, but only its shape.

It is evident that both M and K are highly conditioned by the correct representation of the geometry and by the accurate mass and stiffness distribution along the structure. Table 2 summarizes the computed mass and the overall dimensions for both models. By inspecting Table 2, it is clear that the application of the proposed technique produces a finite element model that accurately describes the building geometry and its mass distribution.

**Table 2:** Mass, overall dimensions and center of mass height.

Model	Mass	Max Dimensions	$h_{g}$
	(tons)	{L×B×H} (m)	(m)
**CAD**	3055.78	9.97 × 9.97× 30.64	13.67
**Voxel**	3032.11	9.90 × 9.79 × 30.60	14.07

Table 3 collects the obtained results in terms of computed frequencies and computed errors. It appears that, for the first six modes, the computed error is always less than 4%, and it is less than 0.1% for the fundamental modes (Mode 1 and Mode 2).

We limited our discussion to the first six modes according to the structural meaning associated with the frequency and the corresponding mode shape [37]. The tower dynamically acts as a cantilever beam whose fine description can be summarized by two bending modes in each horizontal direction plus a torsional mode and an axial mode [16].

**Figure 13:**
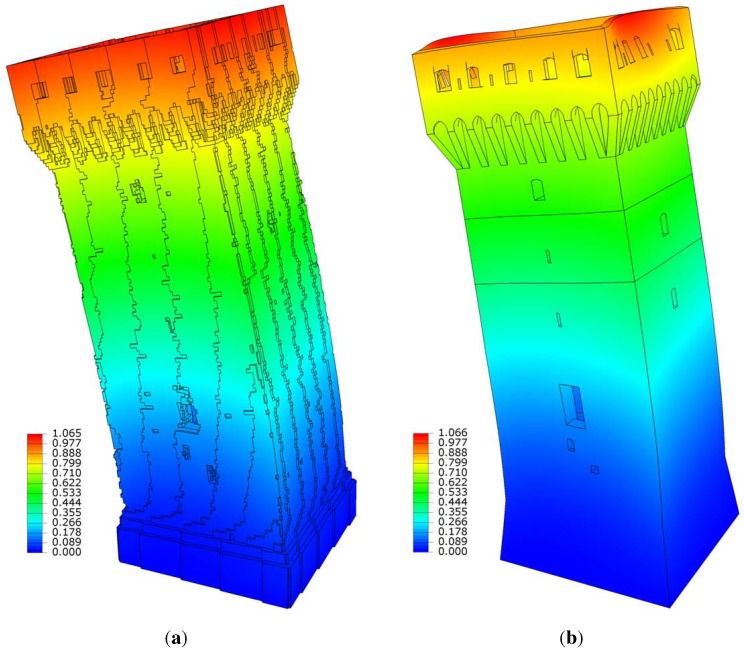
Mode 1: bending mode shape. Displacements magnitude. (**a**) Voxel frequency = 1.9131 Hz; (**b**) CAD frequency = 1.9137 Hz.

Figure 13 illustrates Mode Shape 1, where colors are associated with the magnitude of the computed amplitude (normalized). Mode shapes are in very good agreement as concerns the overall behavior, and they slightly diverge as concerns the local displacement distribution of the top part: in the CAD-based model, some simplistic assumptions have been introduced on this specific part, due to the high complexity of the geometry. Some parts of the structure have been considered adding the corresponding mass values to the model. In this regard, the *z*coordinate of the center of mass has been computed for each model in order to check the overall mass distribution; see Table 2.

**Table 3:** Natural frequencies’ analysis: frequencies of the main mode shapes of the Mastio tower of the San Felice sul Panaro Fortress. Comparison between the voxel-based model and the CAD-based model.

Mode No.	Voxel Frequency (Hz)	CAD Frequency (Hz)	Error (%)	Mode Description
1	1.9131	1.9137	0.031%	1st bending mode (E-W)
2	1.9276	1.9289	0.067%	1st bending mode (N-S)
3	4.5437	4.4253	2.675%	torsional mode
4	7.0804	7.3518	3.692%	2nd bending mode (E-W)
5	7.1654	7.3665	2.730%	2nd bending mode (N-S)
6	8.1623	8.0055	1.959%	axial mode

On the other hand, the voxel-based model captures every single detail of the geometry precisely, thanks to this semi-automatic procedure, avoiding the user interpretation or the necessity to defeature the complexity of the model. This fine description obviously introduces a higher number of degrees of freedom (dof): the voxel model counts 2,237,004 dofs, whereas the CAD-based model enumerates 163,020 dofs. Despite the larger number of dof, the proposed procedure allows one to transform the user time into computational time. Moreover, a more effective FE model prone to optimizing the computational cost, preserving the accuracy, might be obtained by coarsening the resolution of the voxels. The voxel discretization introduces a simplified description of the geometry and leads to a finer finite element model able to precisely capture the geometry features and the corresponding mass properties. The mechanical properties are defined by a punctual characterization, which leads to a very accurate description of the structure, since each voxel can be, generally, automatically associated with a particular property definition, whereas for the CAD-based model each material or property needs a partition of the whole solid model. Further enhancements of the capability to simulate the structural behavior (*i.e.*, introduction of special elements or interface elements) can be easily done manually or automatically through simple selections of elements or nodes, due to the rational database organization, whereas for the CAD-based model, any enhancement of the structural model has to be preliminarily designed along with the geometry.

## 4. Conclusions

A new technique, called CLOUD2FEM, to generate an FE model from a laser scanner survey of a complex building has been presented and applied to study a fortress damaged by the 2012 Emilia earthquake. The development of an FE model obtained using the mentioned procedure has been shown and compared with a CAD-based FE model. The resulting discretized geometry contains all of the information to use with a finite element method procedure, including the mechanical properties associated with the material features, and guarantees the automatic generation of a reliable FE solid model. To assess the accuracy of the proposed FE model, a linear natural frequency analysis has been performed. Results, illustrated by means of computed natural frequencies, show very good agreement.

An estimation of the global time spent to generate the FE model of a complex structure for both CAD-based and CLOUD2FEM-based procedures has been evaluated: we believe that with the use of the proposed method, there is a significant savings of user time. In addition, it should be emphasized that with our approach, also users without advanced structural skills are able to complete the described analysis. We can assert that the proposed procedure is a speedy solution of a complex structure FE model generation problem, especially in the field of cultural heritage, and is furthermore designed to be independent of any particular software.
